# Does local anesthetic temperature affect the onset and duration of ultrasound-guided infraclavicular brachial plexus nerve block?: a randomized clinical trial

**DOI:** 10.1016/j.bjane.2021.02.044

**Published:** 2021-04-03

**Authors:** Ilker Ince, Muhammed Ali Arı, Aysenur Dostbil, Esra Kutlu Yalcin, Ozgur Ozmen, M. Zafeer Khan, Tetsuya Shimada, Mehmet Aksoy, Kutsi Tuncer

**Affiliations:** a Ataturk University School of Medicine, Department of Anesthesiology and Reanimation, Erzurum, Turkey; b Ataturk University, Anesthesiology Clinical Research Office, Erzurum, Turkey; c Cleveland Clinic, Outcomes Research Consortium, Cleveland, United States; d Ataturk University, School of Medicine, Department of Orthopaedic Surgery, Erzurum, Turkey; e National Defense College, Department of Pharmacology, Tokorozawa, Japan

**Keywords:** Local anesthetic, Temperature, Nerve block, Brachial plexus, Interventional ultrasonography

## Abstract

**Background:**

Infraclavicular brachial plexus nerve block is a commonly performed anesthesiology technique in the upper extremity. Local anesthetics may be administered at different temperatures for both neuraxial and peripheral nerve blocks. We aimed to evaluate the effects of the temperature of the local anesthetic at the time of administration on the onset and duration of sensory and motor blocks in infraclavicular brachial plexus nerve block.

**Methods:**

A total of 80 patients undergoing elective upper extremity surgery were randomly assigned to one of the following groups using a computer-based randomization software; low temperature (4 °C) (Group L, n = 26), room temperature (25 °C) (Group R, n = 27) and warmed (37 °C) (Group W, n = 27). A 1:1 mixture of 2% lidocaine and 0.5% bupivacaine was used as local anesthetic. Infraclavicular brachial plexus nerve block was performed under ultrasound guidance in all patients preoperatively. The onset and duration of sensory and motor blocks were recorded.

**Results:**

Each group had different onset of motor (*p* < 0.001) and sensory (*p* < 0.001) blocks. The duration of motor block was similar between groups (*p* = 221). However, a significant difference was found in the duration of sensory block between group L (399.1 ± 40.8 min) and group R (379.6 ± 27.6 min) (*p* = 0.043). There was no complication related to nerve block procedure.

**Conclusions:**

The administration of the local anesthetic at lower temperatures may prolong the onset of both motor and sensory blocks in infraclavicular brachial plexus nerve block.

## Introduction

Infraclavicular brachial plexus nerve block is commonly used to facilitate upper extremity surgeries.^1^ This technique was proposed to have several advantages over general anesthesia including providing a longer postoperative pain relief and decreased opioid consumption, a lower complication rate, and being more cost efficient.^2^ The disadvantage of single shot blocks is that the duration of the block is highly dependent on the local anesthetic agent.^3^ Also, blocks may have a late onset of action due to the long latency period.^4^ Though a continuous catheter may be used to prolong the duration of effect, it also poses some risks such as increased infection rate and higher toxicity.^5^

Various adjuvants (e.g., epinephrine, morphine, fentanyl, clonidine, dexmedetomidine, buprenorphine, tramadol, dexamethasone, neostigmine, ketorolac, magnesium, sodium bicarbonate, and ketamine) were shown to prolong the duration of the effect of the peripheral nerve block and reduce the latency period, but each of these agents has its own side effects.^5^, ^6^ The effect of pre-warmed local anesthetic solution on the onset of sensory block was shown in epidural and spinal anesthesia in earlier studies.^7^, ^8^, ^9^

Outcomes of studies on intra-articular injection of local anesthetics and infiltration blocks are controversial. The number of studies investigating the effect of temperature of the local anesthetic solution on infraclavicular brachial plexus nerve block is limited. In this study, we aimed to evaluate the effect of the temperature of the local anesthetic at the time of administration on the onset and duration of sensory and motor blocks in infraclavicular brachial plexus nerve block.

## Materials and methods

### Setting and study population

Institutional review board (IRB) approval was obtained for the study (B.30.2.ATA.O.Ol.00/137). The sample size was calculated using NCSS-PAS. The primary outcome of the study was the onset of sensory block. According to study by Heath et al, based on a 2-minutes difference was accepted as significant between the groups and standard deviation of 1.39 and 1.12 minutes in the onset of sensory block of median nerve between study groups, 26 patients per group was calculated to achieve 80% power with an alpha error of 0.05.^4^

**Figure 1:**
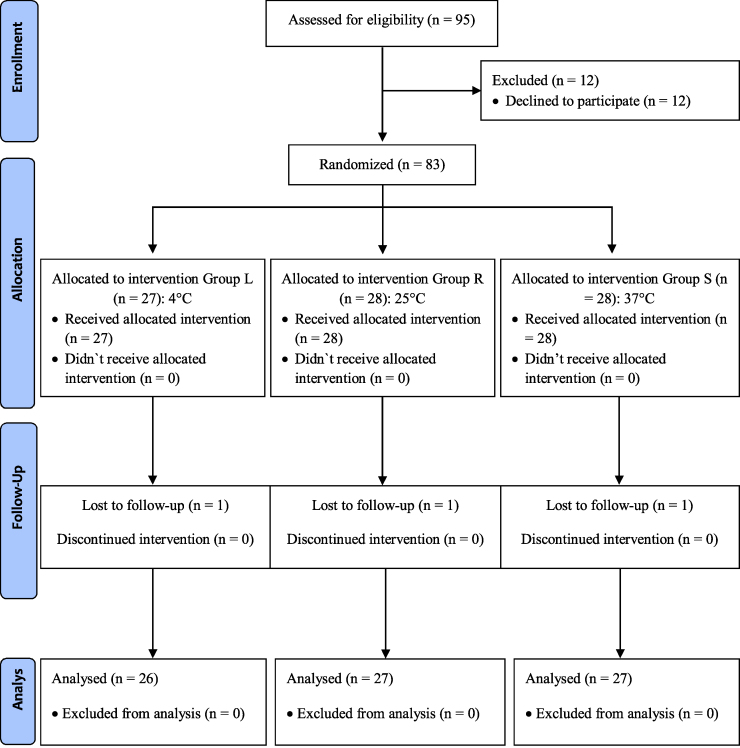
Consort flow diagram.

Patients who were between 18–65 years old, with an American Society of Anesthesiologists (ASA) physical status I–II and undergoing elective upper extremity surgery between August 2016 and August 2017 were included. Patients who declined to participate, having a history of ipsilateral peripheral nerve damage of the upper extremity, bleeding disorder, chronic renal or liver disease, drug allergy, and an active infection at the local anesthetic injection site were excluded. Ninety-five patients underwent elective upper extremity surgery during the study period. Of these, 12 patients declined study participation and 3 patients were lost to follow-up. A total of 80 patients were included in this study (Fig. 1). Patients were randomly assigned to one of the 3 groups using a computer-based randomization software. The local anesthetic mixture was administered to patients in group L (n = 26), group R (n = 27) and group W (n = 27) at 4 °C, 25 °C and 37 °C, respectively (Fig. 1).

Patients were routinely monitored for oxygen saturation, arterial blood pressure, and electrocardiography (ECG) intraoperatively. All blocks were performed preoperatively by an anesthesiologist under the ultrasound (US) guidance (Esaote MyLab30®, Florence, Italy) in a room reserved and equipped for peripheral nerve blocks. The anesthesiologist who performed the blocks was unblinded to the study since it was easily felt the temperature of the local anesthetic during injection it by anesthesiologist. The investigator anesthesiologists who collected to the data was blinded for the study.

After placing the patient in a supine position, a bolus of 1 mg of midazolam was administered via IV cannula (Zolamid® 5 mg/5 mL, Defarma, Istanbul, Turkey), and oxygen was delivered through a nasal cannula. The site around the entry point of the needle of peripheral nerve block was draped in a sterile fashion. The high frequency linear ultrasound probe (Esaote MyLab30® high frequency linear probe) was covered with a sterile ultrasound cover. A 10-cm sonovisible sterile needle (Stimuplex® Ultra, Braun, Germany) was used for the nerve block. Brachial plexus and surrounding structures including axillary artery and vein, and infraclavicular muscles were visualized under US guidance.

Local anesthetic was warmed up to 37 °C for group W, stored at room temperature (25 °C) for group R and stored at 4 °C for group L before use. For each patient, a 20 ml of local anesthetic solution containing a 1:1 mixture of 2% lidocaine and 0.5% bupivacaine was administered. The patients were unblinded for the study since they could feel the cold or warmed local anesthetic during injection.

Sensory block was evaluated using a cold ice pack every 2 minutes. The onset of sensory block was recorded as the time when a total loss of cold sensation on radial, median, ulnar, and musculocutaneous nerve dermatomes was confirmed by the patient. Duration of the motor block was evaluated for each nerve individually using the Lovett Rating Scale at 15 and 30 minutes, 1, 2, 4, 8, 12, 16, 20, and 24 hours. Motor strength was scored between 0 and 6, with 0 representing complete paralysis, 1 almost complete paralysis, 2 impaired mobility, 3 slightly impaired mobility, 4 pronounced reduction of muscular force, 5 slightly reduced muscular force, and 6 normal muscular force. Strength of thumb abduction was evaluated as a reference for the radial nerve motor block, as thumb opposition for the median nerve and thumb adduction for the ulnar nerve.

### Statistical analysis

Data is presented as mean, standard deviation, median, minimum, maximum, percentage, and in numbers. Normality of the distribution of continuous data was tested using Shapiro-Wilk test. In the comparison of continuous variables with more than two independent groups, the ANOVA test was used when the normal distribution condition was met and the Kruskal Wallis test was used when the normal distribution condition was not met. After the ANOVA test, post-hoc tests were performed using the Tukey test when the variances were homogeneous and Tamhane’s T2 test when the variances were not homogeneous. The Kruskal Wallis test was performed using the Kruskal Wallis one-way ANOVA (K samples) test for post-hoc tests. Categorical data was compared using Chi-Square test. Statistical analysis was performed using SPSS v20 (IBM, NY, USA). A *p*-value < 0.05 was defined as significant.

## Results

Demographic data, body temperature, and operative time were similar between groups (Table 1). Mean onset of sensory block in group L, R, and W were 10.9 ± 1.3, 9.6 ± 1.2, and 8.6 ± 1.8 minutes, respectively. Significant differences were detected in the onset between groups (*p* < 0.01), and subgroup analysis revealed a significant difference between each group (Table 2). Mean onset of motor block in group L, R, and W were 13.1 ± 2.2, 11.6 ± 1.5, and 9.8 ± 2.2 minutes, respectively. Significant differences were detected between groups (*p* < 0.05), and subgroup analysis revealed a significant difference between each group (Table 2).

**Table 1:** Demographic and operation-related data.

	Group L	Group R	Group W	*p*-value
	(n = 26)	(n = 27)	(n = 27)	
Age (years)	42.8 ± 16.2	36.5 ± 9.4	37.9 ± 11.9	0.176^a^
Weight (kg)	72.1 ± 13.4	72.8 ± 13.4	76.3 ± 13.3	0.464^a^
Height (cm)	169.5 ± 6.9	170.1 ± 8.4	171.4 ± 8.8	0.670^a^
Gender (male/female)	14/12	16/11	21/6	0.162^b^
Body Temperature (°C)	36.4 ± 1.1	36.7 ± 0.4	36.4 ± 0.6	0.412^a^
Duration of Surgery (min)	41.1 ± 19.7	55.2 ± 23.8	63.7 ± 57.1	0.096^a^

Group L: Low Temperature (4 °C) group, Group R: Room temperature (25 °C) group, Group W: Warmed (37 °C) group.

Values are expressed in mean ± SD.

a *p* > 0.05, One-way ANOVA.

b *p* > 0.05, Chi-Square Test.

**Table 2:** Onset of motor and sensory block.

	Group L	Group R	Group W	*p*-value
	(n = 26)	(n = 27)	(n = 27)	
Onset of Motor Block (min)	13.1 ± 1.2	11.6 ± 1.5	9.8 ± 2.2	< 0.001^a^
Onset of Sensory Block (min)	10.9 ± 1.3	9.6 ± 1.2	8.6 ± 1.8	< 0.001^b^

Group L: Low Temperature (4 °C) group, Group R: Room temperature (25 °C) group, Group W: Warmed (37 °C) group.

Values are expressed in mean ± SD.

Significant difference between all groups after post-hoc test.

a *p* < 0.05, Kruskal Wallis.

b *p* < 0.05, One-way ANOVA.

Duration of sensory block in group L, R, and W were 399.1 ± 40.8, 379.6 ± 27.6, 381.8 ± 29.6 minutes, respectively. A significant difference was detected between groups (*p* = 0.043), and a subgroup analysis revealed a difference between groups L and R (Table 3). Duration of motor block in group L, R, and W were 276.5 ± 16.7, 273.8 ± 14.9, and 269.4 2 ± 12.5 minutes, respectively and were similar (*p* = 0.221) (Table 3). No complication was reported due to nerve block during the follow-up.

**Table 3:** Duration of motor and sensory block.

	Group L	Group R	Group W	*p*-value
	(n = 26)	(n = 27)	(n = 27)	
Duration of Motor Block (min)	276.5 ± 16.7	273.8 ± 14.9	269.4 ± 12.5	0.221^a^
Duration of Sensory Block (min)	399.1 ± 40.8	379.6 ± 27.6	381.8 ± 29.6	0.043^b^

Group L: Low Temperature (4 °C) group, Group R: Room temperature (25 °C) group, Group W: Warmed (37 °C) group.

Values are expressed in mean ± SD.

a *p* > 0.05, One-way ANOVA.

b *p* < 0.05, Significant difference between Group L and Group R, after Post-Hoc test, Kruskal Wallis.

## Discussion

A major finding of this randomized controlled study is that the onset of sensory and motor blocks may be delayed when administered at lower temperatures. Effects of temperature on peripheral blocks have been reported in earlier studies, in which local anesthetics were administered at a low, room, or body temperature.^9^, ^10^ However, there is no study to compare the effect of local anesthetic solution administered at low, room, and body temperature on block characteristics. Although potentiation of the action of the local anesthetic by either cooling or warming have been described, to date there is no conclusive evidence showing superiority of one group (cold/room temp/warm) over another.^9^, ^10^

Heath et al found that the onset of effect the subclavian brachial plexus block using 0.5 mL.kg^-1^ of 0.5% bupivacaine was significantly shorter at body temperature compared to room temperature.^4^ We found that the onset of both sensory and motor blocks after infraclavicular brachial plexus block were significantly earlier in the warm group (37 °C), and the duration of sensory and motor blocks were similar.

In a prospective, randomized, doubled-blind study, Sviggum et al investigated the effect of temperature of anesthetic agent in epidural anesthesia (bupivacaine 0.125% with fentanyl 2 μg.mL^-1^ (20 mL initial and 6 mL hourly boluses) on 54 nulliparous laboring women. They administered anesthetic solution at 20 °C and 37 °C. They found that the onset of action was shorter in the warm group (37 °C) and suggested this provided improved analgesia for the first 15 minutes.^11^

Various factors determine the final outcome when physical properties of a drug are studied in vivo. Increasing the temperature causes a decrease in dissociation constant of the drug,^12^ which in turn increases the unionized fraction. This leads to increased lipid solubility and hence, increases the membrane permeation.^11^, ^13^

In another double-blind, controlled trial, Chilvers et al investigated the effect of warming the local anesthetic on the onset of axillary brachial plexus block.^14^ They suggested that injecting the solution close to the axillary artery may possibly increase the temperature of the solution from room temperature to body temperature quickly. Their results opposed to our findings, in that our study showed a significant difference in the onset of both motor and sensory blocks between all groups.

In a study on patients undergoing transurethral prostate resection (TUR-P) surgery, Nazli et al performed spinal anesthesia at two different temperatures and demonstrated that the onset of action of the block (3 mL of 0.5% levobupivacaine solution) was shorter and its duration was longer at body temperature (37 °C) compared to room temperature (25 °C).^9^ They noted that increasing the temperature of the solution resulted in an increase in the unionized fraction of the drug available to penetrate the nerve, and increased the molecular kinetic energy which leads to increased distribution of drug in CSF. The overall effect was an earlier onset of action and sensory block at more levels of medulla spinalis. It is unclear whether their outcome could be translated to the infraclavicular brachial plexus nerve block. Tomak et al reported that compared to room temperature group, spinal anesthesia using an anesthetic solution at 5 °C caused a delayed onset of action, effected fewer sensory levels of medulla spinalis, and had shorter duration of sensory and motor blocks.^15^ They reported a higher success rate of unilateral spinal anesthesia and lower hemodynamic complications in the cold group.

In an interesting study, Dabarakis et al compared the effect of the temperature of the local anesthetic solution (0.25 mL of 3% plain mepivacaine) on pulpal anesthesia.^16^ They reported that there was no difference in the onset of block between 4 °C and 20 °C, while the duration of block was longer in the 4 °C group. They suggested that the vasoconstrictive effect of cooling was responsible for the result. Cooling also decreases the amplitude, and increases the duration and latency of the action potential.^10^ Recovery of inactivated fibers at low temperatures is slow.^17^

Although we found that administering local anesthetic at a higher temperature shortened the onset of both sensory and motor blocks, and this finding was similar in many earlier studies, the clinical importance of this effect is not clear. Also, storing local anesthetic at room temperature is more cost-effective compared to storing it at a higher temperature. Similarly, we detected a significant difference in the duration of the sensory block between L (4 °C) and R (25 °C) groups. Considering that both groups had over 6 hours of action and there was only a 20-minute difference between groups, the clinical significance of this difference is equivocal.

## Conclusions

Warming the local anesthetic solution to body temperature shortens the onset of action of sensory, and motor blocks in infraclavicular brachial plexus block with no effect on the duration of sensory or motor block. This approach is desirable as it shortens the onset by utilizing the physical properties of the solution without unnecessarily exposing the patient to additional drugs.

## Conflicts of interest

The authors declare no conflicts of interest.
